# Providing medical care for undocumented migrants in Denmark: what are the challenges for health professionals?

**DOI:** 10.1186/1472-6963-11-154

**Published:** 2011-06-28

**Authors:** Natasja K Jensen, Marie Norredam, Tania Draebel, Marija Bogic, Stefan Priebe, Allan Krasnik

**Affiliations:** 1 Danish Research Centre for Migration, Health and Ethnicity (MESU), Section for Health Services Research, Department of Public Health, University of Copenhagen, Øster Farimagsgade 5, 1014 Copenhagen, Denmark; 2 Danish Research Centre for Migration, Health and Ethnicity (MESU), International Health Unit, Department of International Health, Immunology and Microbiology, University of Copenhagen, Øster Farimagsgade 5, 1014 Copenhagen, Denmark; 3 Unit for Social and Community Psychiatry, Queen Mary University of London, Newham Centre for Mental Health, Cherry Tree Way, London, E13 8SP, UK

## Abstract

**Background:**

The rights of undocumented migrants are frequently overlooked. Denmark has ratified several international conventions recognizing the right to health care for all human beings, but has very scanty legislation and no existing policies for providing health care to undocumented migrants. This study focuses on how health professionals navigate and how they experience providing treatment for undocumented migrants in the Danish health care system.

**Methods:**

The study was carried out as part of an EU-project on European Best Practices in Access, Quality and Appropriateness of Health Services for Immigrants in Europe (EUGATE). This presentation is based on 12 semi-structured interviews with general practitioners (9) and emergency room physicians (3) in Denmark.

**Results:**

The emergency room physicians express that treatment of undocumented migrants is no different from the treatment of any other person. However, care may become more complicated due to lack of previous medical records and contact persons. Contrary to this, general practitioners explain that undocumented migrants will encounter formal barriers when trying to obtain treatment. Additional problems in the treatment of undocumented migrants include language issues, financial aspects for general practitioners, concerns about how to handle the situation including possibilities of further referrals, and an uncertainty as to whether to involve the police.

**Conclusions:**

The health professionals in our study describe that undocumented migrants experience an unequal access to primary care facilities and that great uncertainties exist amongst health professionals as how to respond in such situations. The lack of official policies concerning the right to health care for undocumented migrants continue to pass on the responsibility to health professionals and, thereby, leaves it up to the individual to decide whether treatment can be obtained or not.

## Background

In the public debate, undocumented migrants are often recognized only as a threat to national states, while other aspects such as the rights of undocumented migrants are frequently overlooked. Although it has been estimated that there may be as many as 1.9 to 3.8 million undocumented migrants in the European Union [1], these people remain invisible to policy makers in many countries [2-4]. Due to the unrecorded nature of the phenomenon, it is difficult to establish its exact extent; however, it has been estimated that up to 5,000 undocumented migrants may be residing in Denmark [5,6]. Currently, regular immigrants constitute 7,5% of the Danish population corresponding to approximately 420,000 immigrants and 61% of the immigrants are from non-western countries [7]. In the 1960s and early 1970s, immigration into Denmark was primarily dominated by workers from Turkey and Pakistan. In 1973, an immigration stop was introduced meaning that immigration in the 1980s and 1990s primarily consisted of persons who were family reunificated and refugees from conflicts in the Middle East, Sri Lanka, Somalia, and the Balkans [8]. In the last ten years, Denmark has seen a decrease in the number of people who enter the country as asylum seekers and through family reunification whereas immigration of high-skilled labour migrants has increased [9]. The perception of immigrants in Denmark is complex. Many politicians have been working towards more restrictions in the immigration area [10] and, accordingly, Denmark demands some of the highest requirements in the European countries regarding family reunifications and obtaining citizenship [11]. However, sociological investigations of attitudes of the general population show that the Danish population have become more and more tolerant towards immigrants during the last 30 years [10]. To our knowledge no data describing sociodemographic characteristics of undocumented migrants in Denmark exist. Data from Norway and Sweden show that the group of undocumented migrants for a large part consists of rejected asylum seekers and on these grounds it is assumed that this is also the case for Denmark [12].

Denmark has a universal health care system financed through taxes and it covers all persons registered with the National Register of Persons [13]. The majority of services such as visits to the general practitioner, specialists in private practice and hospital treatment is provided free of charge, whereas dental treatment and procurement of medicines and glasses require co-payment [13,14]. A health insurance card is issued to all persons officially residing in the country by the municipality of residence and serves as a proof of entitlement to care. The health insurance card must be presented when obtaining treatment and all contacts with the health care system are registered under a person's social security number. The hospitals are owned and managed by the regions, whereas general practitioners are self-employed, but still receive the majority of their funding from the regions through a combination of fee for service and a fixed fee from each person enrolled in their practice [15]. Extensive care is offered to persons officially recognized in the system, but there has been very little focus on undocumented migrants' access to care.

Denmark has ratified several international conventions recognizing the right to health care for all human beings; however, official policies in the area of access to health care services for undocumented migrants are scanty. Currently, the only explicit reference relating to entitlement to health care services for undocumented migrants is in Paragraph 42a of the Danish Alien Act, which states that undocumented migrants registered with the authorities and taking residence in an asylum centre will acquire rights to health care on the same terms as asylum seekers [16]. In practice, this will imply that the undocumented migrant will have to apply for asylum or leave the country and, therefore, it is not a realistic option. Access to health care treatment in the general health care system is very restricted for undocumented migrants. Due to a lack of health insurance card and registration with the National Register of Persons, undocumented migrants will have no formal access to general practitioners in the general health care system. Under normal circumstances, the health insurance card must also be presented to obtain treatment in hospitals. However, the hospitals are assigned an "acute obligation" implying that foreign citizens without residence in Denmark, but who are temporarily staying in the country, have the right to emergency hospital care on the same terms as people residing in the country in case of an accident, sudden disease and birth or deterioration of chronic disease [13,17]. Furthermore, additional treatment can be provided to persons without residence if, in the present circumstances, it is assessed that it is not reasonable to refer the person for treatment in the home country. The regional authorities are formally responsible for deciding whether it is reasonable that such services are delivered free of charge, but services may be subjected to payment [12,13,17].

In general, the regulations concerning undocumented migrants' access to health care are inadequate and inexplicit. As the right to health care of undocumented migrants is not explicitly dealt with in the health care legislation the interpretation of the legislation can give rise to problems. Therefore, the responsibility of deciding whether an undocumented migrant should obtain health care treatment is, in practice, left to the individual health professionals, who have no guidelines to which they may refer. This study is the first to explore how health professionals in the Danish health care system view access to care for undocumented migrants and how these professionals navigate in a system without clear guidelines regarding health care for undocumented migrants.

## Methods

The study is part of an EU-project on European Best Practices in Access, Quality and Appropriateness of Health Services for Immigrants in Europe (EUGATE) conducted in 16 European countries [18].

The Danish data were collected September 2008-January 2009. We conducted 15 interviews, distributed as follows: Nine with general practitioners (GPs), three with physicians working in emergency rooms (ER), and three with managers of psychiatric residential units. Participants were selected from three different hospital catchment areas with high proportions of immigrants and located in urban areas in the vicinity of Copenhagen. The interview guide developed by the EUGATE coordinating group was translated and adapted to Danish conditions. The first part of the interview focused on immigrants in general. During the interview health professionals were then asked to answer questions on three case vignettes with 1) an undocumented migrant 2) a refugee and 3) a labour migrant.

This article builds on the 12 interviews with GPs and ER physicians, and analyses their experiences in treating undocumented migrants in Denmark. Nine out of 12 participants were women and two of the participants were immigrants whereas the rest were of Danish origin. The participants all had experience of treating undocumented migrants. One GP mentioned that she had not had any undocumented migrants in her practice yet. Since she was still familiar with the way providing treatment in general practice is organised and was able to assess limitations in providing care for this migrant group based on her experiences from other parts of the healthcare system, her statements were included in the analysis. Data from the psychiatric residential units were excluded from the analysis on undocumented migrants, given that none of the participants had any experience with this group of immigrants and, accordingly, could give only hypothetical answers. The vignettes on undocumented migrants presented to the health professionals varied slightly according to the type of service (Table 1), but the discussion points used as a basis for the interview were the same (Table 2). The interviews were analysed using qualitative content analysis as described by Graneheim and Lundmann [19]. In the process, focus was on both commonalities and traits specific to certain service providers. In this article undocumented migrants are defined as migrants without a valid residence permit authorising them to regularly stay in the country in which they are currently residing. The routes into becoming undocumented are many, and the term covers migrants who may have been unsuccessful in the asylum procedure, have overstayed their visa, or may have entered the country through irregular means [20]. It can be added that undocumented migrants is a flexible category as migrants who enter a country through regular routes with a valid visa, may later find themselves in a situation with irregular migration status and migrants who enter through irregular means may later become regularised [21].

**Table 1:** Vignettes used for GPs and ER physicians

***GPs***
A male, 28 years old, coming from Ukraine, presents with pain when urinating and has a slight fever. He does not speak any language that the doctor understands. He has no insurance, no identification and no residence permit.
***ER physicians***
The patient arrived in the host country as an undocumented migrant about 1 year ago. He is 25 years of age and of Ukrainian origin. He does not speak any language that the ER staff understands and presents with an intense lower abdominal pain.

**Table 2:** Discussion points for the vignettes on undocumented migrants

• From your perspective, what are the differences, if any, in the treatment for this patient compared to a patient with a similar condition from the native-born population?
• From the perspective of a patient, what do you think are the specific problems this patient would encounter that are different from those of a patient with a similar condition from the native-born population, and how would they be overcome?
• What are the specific further pathways and treatment options, if any, for this patient that are different from those of a patient with a similar condition from the native-born population?
• Would you inform the police and/or other authorities?

## Results

The themes emerging in the interviews with GPs and ER physicians on their experiences with treating undocumented migrants and these migrants' access to health care services are presented below. An overview of the results is presented in Table 3.

**Table 3:** Main findings of the study

**Access to ER care**
• ER physicians reported no difference in admission or treatment of undocumented migrants
• Lack of access to previous medical records was a problem for ER physicians
• Delay in treatment seeking implied that undocumented migrants presented with more advanced disease
• Providing treatment for undocumented migrants increased the administrative work.
**Access to GP care**
• Lack of formal entitlements to primary care made access more difficult
• If the general practitioner agreed to treat the undocumented migrant, there was no difference in the services they offer in their own clinic
• Referral pathways are more complicated for undocumented migrants e.g. only access to external diagnostic facilities at the ER
• Follow-up was problematic due to lack of continuity of care for undocumented migrants.
**Language issues**
• Diagnosing was complicated due to language problems
• Language barriers made it difficult for the health professional to provide psychosocial support to the undocumented migrant.
**Strategies for solving financial issues for GPs**
• GP covered expenses of treatment
• GP offered treatment at a charge.
**Uncertainty of the health professionals**
• GPs were uncertain concerning hospital admission of undocumented migrants
• GPs were uncertain about whether they could prescribe medicine because the undocumented migrant had no social security number
• The encounter with undocumented migrants could evoke unpleasant feelings in GPs
• Health professionals did not know whether they were obliged to report undocumented migrants to the police.
**Involvement of police or other authorities**
• Health professionals choose to focus solely on the medical problem of the undocumented migrant
• Health professionals would report the undocumented migrant to the police only when they suspected a serious crime was involved
• ER physicians would contact the police only if it was necessary for identification of the undocumented migrant or relatives in the case of a fatal outcome of treatment.

### Access to emergency care

The ER physicians all expressed that the medical treatment of undocumented migrants would not differ from that of any other person attending the ER. "*There is no difference at all for a person coming in with abdominal pain and who can show themselves that it hurts and you can tell it hurts when you put pressure on it. The examination programme will be the same. The patient in question could just as well be a person without a language. You may have a deaf patient and sometimes we have patients who are mentally disabled and who are not able to communicate to the extent we are, and they will all be examined in the same way*" (ID 41, female ER physician). If necessary, it is also possible to admit the undocumented migrant to the hospital "*There is no difference. If there is a need for admission or further diagnosing this will take place on the same terms as for everyone else. I am not familiar with anyone whom has been rejected. You'd probably deal with that later*" (ID 40, male ER physician).

The ER physicians expressed that lack of access to previous medical records and lack of contact persons could be a problem when treating undocumented migrants. The lack of a contact person is described as particularly important by the ER physicians in the case of a fatal outcome for the undocumented migrant. One ER physician said that undocumented migrants often delay seeking treatment, implying that they already are in a more vulnerable situation prior to the initiation of treatment due to more advanced disease. Although the ER physicians explained that undocumented migrants could be treated and admitted without problems, they expressed that it increased the administrative work since the undocumented migrant was not registered in any official systems. A replacement social security number is created to be able to enter them into the hospital IT system. This procedure is also employed in other situations.

### Access to primary care

Contrary to the ER physicians, the GPs explained how the undocumented migrants would face administrative barriers when trying to access treatment in their practices. The administrative barriers for undocumented migrants include lack of health insurance card and derived problems of getting past the administrative personnel to access treatment. One of the participants said "*He would be met with the necessity of having to show his health insurance card at the reception desk, and if he didn't have that, then he'd have to show the insurance he has that entitles him to treatment free of charge. If he didn't have any, my staff would come and ask one of us doctors how to deal with it. Possibly, he would feel under suspicion. In any case, he wouldn't experience that it was as easy to get access as a native-born Dane or an [regular] immigrant would*" (ID 52, female GP). Though the GPs generally express that they will provide treatment for an undocumented migrant there may be a difference between individual health professionals as to how willing they are to offer treatment. One of them expressed it in the following way "*We normally do not treat people who do not have a health insurance card unless it is very special circumstances. Otherwise we tell them to contact the ER*" (ID 54, female GP). If the GPs agreed to treat the undocumented migrant, the majority of participants expressed that there would be no difference between an undocumented migrant and a regular patient concerning the medical treatment the GPs can provide in their own practice "*I'd have to make the diagnosis here. I couldn't send a blood or urine sample for cultivation. There are a lot of things you can't do when you don't have a health insurance card and may not even be allowed to be here. Then I'd have to use my clinical sense (...). I could ask him to produce a urine sample and I have my own microscope so I could get quite far on my own*" (ID 51, female GP). However, as it is also expressed in the above quote the problems arose when external diagnostic facilities needed to be involved. The participants expressed that they could not access facilities such as X-ray, ultrasound, laboratories and specialists in private practice due to the lack of a social security number. Therefore, the only official solution would be to refer the undocumented migrant to the ER if further diagnostic procedures were required. Many GPs expressed that they would choose this option instead of a specialist in private practice. Some GPs described different strategies of how to circumvent official barriers in the health care system; one GP explained how she had used personal contacts with specialists in private practice to be able to refer undocumented migrants to further treatment. Others mentioned the possibility of sending in tests in their own name or creating a fake social security number but emphasized that in reality they did not know how they would handle the situation.

The GPs described how the undocumented migrant would have to settle for less than would be the case if a native-born Dane were treated and that the treatment to some extent would be based on assumptions "*If I couldn't get a proper anamnesis, then I would have to treat him based on the fever and that it hurt when he urinated and then think that it was probably a urinary tract infection. Men don't get urinary tract infections very often so I would have to safeguard for various bacteria, so his treatment would be very broad compared with others' *" (ID 49, female GP). A few GPs also expressed that follow-up would be problematic as they might not be able to contact the undocumented migrant if any severe illness were discovered in the test results.

### Language issues

The problem of language is expressed by both ER physicians and GPs, who describe how language barriers pose problems, both for themselves and the undocumented migrants. The health professionals' work is complicated because the undocumented migrant cannot explain the problem, which makes it more difficult for the physician to reach a diagnosis. At the same time, health professionals described how it must be stressful for the undocumented migrants not to understand the medical problem and the health professional would not be able to reassure him/her due to the lack of a common language. "*It would be a problem that the communication was poor. If he showed up, one would assume that he had an acute need, that he was in real pain, or he thought that it was life threatening. Not being able to get a proper explanation or have his worries interpreted would add to his anxiety and fear*" (ID 48, male GP). Health professionals suggested several solutions as to how to handle the communication problem, such as trying to arrange for a formal interpreter or a bilingual employee to attend, making use of the undocumented migrants' informal networks to arrange someone to interpret, or simply by using non-verbal communication.

### Financial aspects

Financial issues were solely mentioned by GPs. However, for this group it is an important aspect, and it is mentioned by all the GPs interviewed. The practitioners expressed very different strategies concerning how to approach payment for services. Some GPs expressed that they would treat the undocumented migrant free of charge and one said "*In my own practice, I work for free if undocumented migrants show up, I can't really send the bill anywhere. But it isn't a problem, we can handle that. It is part of the medical pledge to treat people, and that can't be accounted for in money*" (ID 46, female GP). Others explained how they would be willing to offer help, but that they would charge for the services "*Then he would have a financial perspective where I would always make him relate to 'who is paying for this' and he would probably get nervous about that as well. I couldn't send him to a laboratory without him being required to pay. Naturally, I would try to handle it without any big expenses, but to some extent he would certainly be pressured financially*" (ID 48, male GP). However, the majority of responses fell in between these two statements. Many GPs expressed that given the undocumented migrant were able to pay, it would not be a problem to obtain treatment from the GP, but if he/she were without means, the majority were uncertain of whether they would demand payment and expressed that it would depend on the situation. Additionally, the GPs expressed that problems would arise if they had to involve finances outside their own practice, for example, the use of laboratory facilities or prescriptions for medicine. Several GPs expressed that they would not know how to handle such a situation, and a female GP explained how she would simply refrain from referring the undocumented migrant because she would not be able to assess the financial consequences of this action on his/her behalf.

### Uncertainties of health professionals

Several GPs expressed uncertainty about how they would handle the situation of treating an undocumented migrant. As mentioned, this uncertainty could relate to whether to demand payment for treatment, but also arose in many other situations, such as whether a patient could be admitted "*(...) if he were Danish you would admit him to the hospital, but you couldn't do that. Or could you? No... and maybe he would also say no. If he were Danish you may, medically speaking, do the correct thing by admitting him. You could get in trouble with the complaints board by just sending him home with some pills. But for this man, you might complicate things further by admitting him (...). A guy like him would probably not be interested in being admitted, I don't know*" (ID 51, female GP). Problems also arose in the case of having to prescribe medicine. Some GPs were hesitant as to whether they would be permitted to prescribe medicine for the undocumented migrants. One GP expressed that the undocumented migrant may not wish to have a prescription made out in his/hers own name; another expressed doubts about whether it would be at all possible for the undocumented migrant to purchase medicine at a pharmacy. In addition, one GP expressed that treating undocumented migrants would make him feel uncomfortable "*Normally, I would say that I would probably feel quite uncomfortable with such a patient. As I said, it would provoke many thoughts. Why are you here illegally? And what was it... Ukraine? There's no war or anything in Ukraine now - even if there were a war they could apply for asylum*" (ID 53, male GP).

### Involving police or other authorities?

The ER physicians and GPs interviewed expressed in general that they would not involve the police or other authorities to report an undocumented migrant who contact them regarding treatment. They expressed that medical treatment was something that needed to be kept separate from the legal status of the patient: "*Actually, I don't care about people's legal basis for staying in Denmark; if they are sick and I am a doctor, then I am obliged to treat them according to the Hippocratic Oath*" (ID 49, female GP). Other participants explained how they would simply regard undocumented migrants as patients, irrespective of what their legal status might be, or how they would choose simply to focus on the medical side of the situation and ask about as little else as possible. Although the ER physicians and GPs agreed that they would not report an undocumented migrant to the police, several participants expressed that they did not know whether they were allowed or possibly even supposed to do so. One GP said "*I don't think so. Not unless I am obliged to do so, but I don't know. It hasn't happened to me yet-no undocumented migrant has turned up here, but I don't know. I actually don't know whether you are obliged to do so, but I don't think you are as a doctor. I have treated quite a lot in the ER, and we didn't call the police then. We just treated them and sent them on. So I don't expect that you are obliged to do so, so no, I wouldn't*" (ID 49, female GP). Some physicians were more certain of their actions, basing their rationale behind this on the medical profession "*No. I don't imagine any doctor would. After all, we do have a feeling of a medical class and all people need to be able to see the doctor without being scared. We see it as a holy place. There have been many discussions over the years concerning patients who enter the ER, and therefore I ask as little as possible and make sure to stick to the purely medical side of things*" (ID 51, female GP). However, many participants mentioned special circumstances that would merit contacting the police. Such circumstances were not related to the patient being an undocumented migrant; they were related to concern about whether the patient was involved in a crime and whether the lives of other people were at stake or whether identification would be necessary. Furthermore, two physicians from the ER departments expressed that contacting the police might be necessary for identification of the patient and relatives in the case of fatality during treatment.

## Discussion

### Methodological considerations

The health professionals included in our study were selected in urban areas with high proportions of immigrants in the vicinity of Copenhagen. Therefore, we expect that they were also from areas with a high exposure of undocumented migrants. It is possible that a relatively frequent contact with undocumented migrants influenced how the health professionals in this study viewed the access to care for this group of patients. Frequent exposure to a vulnerable group of patients in severe distress may lead to stronger feelings of sympathy; nevertheless, it may also spark negative feelings in health professionals if they feel there is an imbalance in the way the individual or institutional resources are spent. The delivery of health care services to undocumented immigrants is a sensitive topic. The fact that the interviews also focused on immigrants in general meant that there was time to establish trust between the interviewer and the health professional before the issue of undocumented migrants was approached. In addition, only the interviewer and interviewee were present during the interviews, which helped to secure a confidential atmosphere. The ER physicians in our study describe that there is no difference in access to treatment for undocumented migrants in the ER. This may be explained by the simple fact that the physicians included in our study were willing to treat undocumented migrants without any consideration of their legal status. The ER physicians were from three different hospitals, but it is possible that other physicians working in the same ERs may have held different views towards treating undocumented migrants than the ones included in our study. Another explanation may be that we interviewed only those last in the chain of providing care, namely, the physicians. When entering the ER, the first step is to consult the registration desk and then the initial medical visitation is carried out by nurses. Therefore, it is possible that undocumented migrants are denied access to treatment before they even get in contact with a physician and negative attitudes among administrative personnel or physicians may act as an important barrier in access to care. Lastly, if physicians actually held the view that undocumented migrants are not entitled to care, they might not have been willing to admit this to the interviewer as this would conflict with their medical pledge and considerations of social desirability in answers provided during the interviews may have been present. This could imply that our results provide a more positive picture than the reality experienced by undocumented migrants trying to obtain ER health care and, therefore, it is important also to investigate how the group of undocumented migrants themselves experience access to health care services. Furthermore, conducting interviews with physicians and nurses from other hospital wards that to a higher extent provide elective treatment may also lead to different views on access to health care for undocumented migrants.

### Access to care

According to the findings from our study, access to general practitioners is a particular problem for undocumented migrants. The most common barriers described in accessing treatment relate to administrative barriers, restricted possibilities of further referral and financial problems. Similar problems have been described in a study from Canada exploring access to health care services for undocumented migrants. It is reported in this study that administrators turn away undocumented migrants due to a very strict application of administrative procedures, and that health professionals in primary care settings are forced to work around institutional procedures, for example, by patients coming directly to their office instead of presenting at the front desk and by processing patients without keeping records of the action [22]. In our study the ER physicians reported that there were no difficulties in accessing emergency care facilities for undocumented migrants. However, this is not in line with findings from a recent study based on interviews with undocumented migrants themselves. In that study, several incidents of undocumented migrants being refused care in the ER are reported, and it is concluded that the physicians are largely unaware of the undocumented migrants' rights to emergency care [23]. This indicates that access to emergency care for undocumented migrants may be highly arbitrary and depend on the individual physician. Our findings from the ER are based solely on interviews with physicians. Typically, a selection of patients will be made by either a nurse or the administrative personnel in the hospital before there is any access to a physician; therefore, it is highly likely that undocumented migrants encounter barriers in access to treatment before reaching the individual ER physician. Hospital administrators are often the first point of access in the hospital, and they are not bound by professional ethics in the same way as nurses and physicians. Therefore, administrators may not face the same problems in turning away undocumented migrants at the hospital reception desk [24].

Studies from several other countries have shown that undocumented migrants experience obstacles in access to care [25-29]. Poor access to care will not only have serious health consequences for undocumented migrants and public health in general [24,25,28], but may also lead to problems for health professionals when providing care for these migrants as their options of providing proper treatment and possibilities of referral for this group of migrants is restricted.

### Quality of care

The GPs in our study described how undocumented migrants would have to settle for less treatment as compared with that of a native-born Dane because they would have no formal access to further diagnostic facilities and very restricted possibilities of follow-up care. Lower quality of care for undocumented migrants has previously been described [28,30]. In a recent study, conducted in a clinic for undocumented migrants in Berlin, it is reported that the care of pregnant women and infants is insufficient. Many undocumented women do not present themselves at the clinic until the final trimester or simply never return to obtain test results, rendering any preventive services offered by the clinic unfeasible. In addition, it is reported that undocumented migrants have difficulties accessing a regular supply of medication for chronic illnesses, that there is a lack of mental health care options for undocumented migrants and that they experience difficulties accessing emergency care and have a delay in presentation [28]. It is also highlighted by one of the physicians included in our study that undocumented migrants have a delay in seeking medical care; accordingly, they are in a more vulnerable position initially. The concern regarding undocumented migrants presenting late and in a more severe state of health is frequently reported in other studies as well in primary care settings and hospitals [28,31-34]. The GPs in our study reported that they had contact with this group of patients but were not able to offer optimal treatment due to restrictions in the health care system.

### Consequences for health professionals

The findings of this study suggest that particularly GPs experience problems and uncertainties in relation to treating undocumented migrants. There is the financial consideration that providing care for undocumented migrants may result in an economic burden on the individual GP, because the GP would not be able to have the expenses reimbursed through the national health insurance, or there is the risk that the undocumented migrant is denied care if he or she cannot pay.

Although the health professionals included in our study were generally positive towards treating undocumented migrants, the provision of care is in the margins of the Danish health care system as providing care for this patient group may take place secretly and without official registration in the health care system. In accordance with our findings, the health professionals in the previously mentioned Canadian study also describe how they provide care for undocumented migrants by circumventing the system. While this is tacitly accepted by some managers, others have been found to encourage their employees to refuse services to undocumented migrants. Considerable frustration arises from constantly having to work around the system to provide care for patients the health professionals feel have a right to care [22,31]. In addition, treating undocumented migrants discretely or in hiding will continue to isolate both patients and health professionals from other team members, and it may burden individual physicians willing to take on the responsibility of providing care [31]. A physician working in a maternity ward in the Canadian study explained how she would have to give up her spare time to assure that undocumented women could deliver at the hospital because her colleagues did not share her opinion of undocumented women's rights to health care [22].

Access to health care for undocumented migrants is one of the least regulated areas in the Danish health care system as it is not explicitly recognized in Danish legislation and policies [35]. The lack of official recognition of undocumented migrants' right to health care may lead to substantial concerns among health professionals about how to handle such encounters. This is clearly reflected in the interviews with the GPs in our study, and it places heavy responsibility on the individual health professional when having to navigate access to treatment. It has been described by American authors how nurses may encounter ethical dilemmas in treating undocumented migrants due to conflicting principles. Providing care while remaining within the restrictions of the law may conflict with ethical principles such as non-malfeasance and beneficence [36]. Likewise, it is reported in other European countries that professional groups experience clashes between what their professional ethics dictate and the incriminatory discourse regarding undocumented migrants [24]. Health professionals may find themselves in situations where they have to balance their medical pledge against financial considerations of their own or their institution and the unclear legal rights to health care for undocumented migrants. Recently, the ethical dilemmas for health professionals and the right to treatment for undocumented migrants have started being debated openly in medical circles [12,37,38]. In addition, the Danish Medical Association, the Danish Red Cross and the Danish Refugee Council is advocating for public health care services for undocumented migrants and they are now taking the initiative to start up a clinic during the next few months offering health care services to undocumented migrants in Copenhagen [12,39]. However, in lack of a response from the Ministry of Interior and Health the clinic will be run on a voluntary basis to start with [39] and has so far received great support from health professionals signing up to volunteer [40,41].

### Implications of legislation and policy

Access to health care for undocumented migrants varies with national legislation and policy, and other European countries recognize the right to health care for undocumented migrants in their legislation to a much higher extent than in Denmark [26]. For instance, Spain implemented legal changes in 2002 that grant all undocumented migrants the right to medical cards and free medical care on the same terms as regular migrants and citizens [26,42]. However, the legal changes are not without problems as undocumented migrants are still subjected to certain preconditions to be registered with the municipality [24] and the police are allowed access to the local registers [26]. This implies that many undocumented migrants will not register and consequently have no access to health care in practice [26]. Despite the conflicting legislation, it is reported in a study conducted in 2005 that there is no difference in the use of health services between undocumented and regular migrants after the introduction of these legal changes [42]. In a previous Spanish study based on the same methodological approach, but before the adoption of the mentioned legal changes, it was concluded that having sought care during last episode of illness was more than three times as likely among immigrants with regular status than among undocumented migrants [43]. Although undocumented migrants may still experience other barriers in access to health care services, granting them official rights to health care is a respect of basic human rights and a significant political statement in that their existence in the country would officially be recognized.

As in Denmark, the national legislation regulating access to health care for undocumented migrants in Sweden is limited. Access to health care for undocumented migrants is regulated by the Health and Medical Care Act, which describes that county councils should provide treatment for all persons in need of "immediate health care" [24,44]. This provision is only indirectly applicable to undocumented migrants and access to care is very restricted based on the national legislation [24,45]. However, Sweden has seen regional initiatives regarding formulation of policies in the area of provision of health care for undocumented migrants. The extent of coverage and the emphasis on payment for services greatly varies, but the vast majority of county councils have formulated some sort of policy regarding access to health care for undocumented migrants [45]. Although it is still the individual health professional who decides whether an undocumented migrant will access treatment, the existence of an official policy is a support for the health professional in the concrete situation [45,46].

The scanty legislation regulating access to health care for undocumented migrants in Denmark implies that access to and extent of medical care for this group is highly dependent on the individual physician the patient encounters. In 2003, this led to a request to the National Board of Health concerning obligations regarding how to act in situations when presented with false identification papers for the national health insurance. The response letter was sent to leading health inspectors and specifies that in an emergency situation physicians are obliged to initiate the best possible treatment regardless of legal status and that undocumented migrants are entitled to an assisted delivery. However, it is also stated that the same obligation does not hold for elective treatment to the same extent, but nothing further is specified on this matter [47]. Neither are financial implications for GPs touched upon, despite this being found of great importance in our study.

### Human rights

The dominant discourse amongst authorities in developed countries is that irregular immigration is a criminal activity, and undocumented migrants are people who have tried to gain advantages to which they are not entitled. This view leaves little room for sympathy for undocumented migrants when national authorities deny them access to social benefits and other protection [3]. However, Denmark has ratified several human rights instruments stating that health care is to be provided for every human being, upholding the Declaration of Human Rights, which states that every human being is entitled to a living ensuring his or her right to health and medical assistance as well as care for mothers and children [48], The International Convention on the Elimination of all Forms of Racial Discrimination states that all human beings are equal in the law in relation to health [49], the International Covenant on Economic, Social and Cultural Rights states that all human beings are entitled to enjoy the highest attainable level of physical and mental health [50], and the European Social Charter recognises the right to protection of health [51]. Denmark acknowledges general principles of equal access to health care services; however, the findings of this study show that health professionals remain alone with their uncertainties about how to act in concrete situations when encountering undocumented migrants.

## Conclusions

Based on the findings of this study, it is evident that health professionals consider that undocumented migrants experience an unequal access to primary care facilities, and that substantial uncertainty exists amongst health professionals concerning how to respond when providing care to undocumented migrants. However, there are no official instructions regarding how health professionals should respond to the health needs of undocumented migrants. The lack of official policies on the delivery of health care to undocumented migrants will continue to result in a series of dilemmas for the individual health professional. In future studies it would be relevant also to investigate how nurses and administrative personnel, working both in ERs and with GPs, view access to health care for undocumented migrants as these professional groups are the entry point to access treatment.

## Competing interests

The authors declare that they have no competing interests.

## Authors' contributions

NKJ has made substantial contributions to the design of the study and has carried out the data collection, the data analysis, and drafted the manuscript. MN and TD have contributed to the analysis and interpretation of data and have revised the manuscript critically for important intellectual content. SP, MB and AK has made substantial contributions to the design and has revised the manuscript critically for important intellectual content. All authors have read and approved the final manuscript.

## Pre-publication history

The pre-publication history for this paper can be accessed here:

http://www.biomedcentral.com/1472-6963/11/154/prepub
